# Segmental Bioimpedance Analysis as a Predictor of Injury and Performance Status in Professional Basketball Players: A New Application Potential?

**DOI:** 10.3390/life12071062

**Published:** 2022-07-15

**Authors:** Alexander Bertuccioli, Marco Cardinali, Piero Benelli

**Affiliations:** 1 Department of Biomolecular Sciences, University of Urbino Carlo Bo, 61122 Urbino, Italy; piero.benelli@uniurb.it; 2 Department of Internal Medicine, Infermi Hospital, AUSL Romagna, 47921 Rimini, Italy; marco.cardinali@auslromagna.it

**Keywords:** bioimpedance analysis, BIVA, segmental bioimpedance analysis, basketball, injury predictor, performance predictor

## Abstract

Bioelectrical impedance vector analysis (BIVA) is a technique used for the assessment of body composition based on the electrical properties of biological tissues and for evaluating variations related to hydration and nutrition status changes. The present study aimed to investigate the possibility of predicting performance status and injuries using segmental BIVA analysis. Data were collected from 14 professional male athletes aged between 20 and 39 years of Caucasian and Afro-American ethnicity belonging to the US Victoria Libertas Pallacanestro Pesaro team in the Italian Serie A basketball championship. From an analysis of training injuries, the data highlight a possible positive link between the number of training injuries and upper hemisoma reactance (XCEmsSup) (t = 2.881, *p* = 0.007), an inverse relationship between training injury duration and higher right lower limb reactance (XCLegDx) (t = −4.213, *p* < 0.001), and an inverse relationship between injury duration and higher body mass index (t = −4.213, *p* < 0.001), highlighting how higher cellularity seems less prone to severe training injuries. Analyzing match-day injuries, right upper-limb higher reactance (XCArmdx) negatively correlates with match-day number of injuries (t = −4.469, *p* < 0.001), right upper limb resistance (RZArmDx) negatively correlates with lower match-day injury duration (t = −4.202, *p* < 0.001), and trunk resistance (RZTrunk) positive correlates with lower match-day injury duration (t = 2.803, *p* = 0.008), in contrast with the training data analysis. Analyzing the relationship between the BIVA parameters and performance indicators, right upper limb resistance (RzArmDx) has a positive link with plus–minus (t = 2.889, *p* = 0.007); however, RzArmDx negatively correlates with assist number (t = −3.362, *p* = 0.002), and BMI is directly proportional to assist number (t = 2.254, *p* = 0.032). These first data suggest a good correlation between the cellularity of different body districts and the risk of injuries in training but still leave several doubts surrounding the concrete predictive potential regarding performance and injuries during competitions while considering the numerous factors involved. Further studies on BIVA and similar applications could provide tools for managing athlete health and physical integrity preservation and potentially help us better understand the factors involved in improving performance.

## 1. Introduction

Body composition assessment techniques are considered useful tools for the evaluation of general health status and are increasingly relevant in clinical practice [1]. Anthropometry is the most commonly used methodology for assessing body composition and can be applied to various tasks, such as assessing states of health (and prognostic indexes) or estimating body functionality [2]. Bioelectrical impedance vector analysis (BIVA) represents a technological evolution that extends and integrates assessments that can be carried out using traditional anthropometric assessment techniques.

BIVA is a technique that was developed in the 1990s for the assessment of body composition, allowing us to evaluate variations that occur subsequent to hydration and nutrition status changes [3]. BIVA is based on the electrical properties of biological tissues. When exposing the human body to a weak alternating electric current, electricity flows along highly conductive body tissues [4]. While a low-frequency current is unable to pass through cells, it is capable of flowing through extracellular fluid [5]. However, the cell membrane acts as a capacitor that stores and releases electrons at a high frequency, producing a current. Thus, it could be said that current flows through cell membranes and tissue fluids [6]. Body water volume represents the width of the passage through which electricity flows and is represented by impedance. Bioimpedance—or biological impedance—is defined as the ability of biological tissue to impede passage of an electric current [7]. BIVA measures resistance (R) and reactance (Xc) values. R represents the opposition to the flow of an alternating current through intra- and extracellular ionic solution, whereas Xc represents the capacitive component of tissue interfaces, cell membranes, and organelles [8,9]. Due to the non-invasiveness, the relatively low cost, and the portability of BIVA, many studies on body composition and clinical condition evaluations have been carried out using this technique [5]. One evolution of BIVA is the study of segmental body composition, a technique that allows for the evaluation, for example, of the lean mass and fat mass of the upper and lower body districts and of the upper and lower limbs, with a much higher potential for detailed information than a total body analysis [10]. This potentially overcomes the limit established by the analytical model of the total body BIA, which represents the human organism as a set of five conducting cylinders with an almost constant resistivity [11]. Among the different possible applications studied for the segmental BIVA, we found an evaluation of body symmetry in an asymmetrical sport in young tennis elite players [12] and an evaluation of post-injury follow-up in high-level team-sport athletes [13].

Injuries are an element of considerable importance in determining the number of games played and, consequently, a player’s performance, including the entire process involved in the recovery and return to full functionality of the athlete.

Analyzing professional athletes in the NBA circuit, Torres-Ronda et al. reported an increase in lost games and single injuries in the period 2020–2021 with a greater involvement of the lower body areas, affecting the knee, ankle, and foot in particular, with the main injuries being of the tendon/ligamentosis (1.16 and 0.21, respectively), followed by muscle components (0.69 and 0.16, respectively) and bone components (0.30 and 0.03, respectively). Most injuries and games lost due to injuries occurred mid- to late season in athletes with experiences between the ages of 6 and 15 [14].

Analyzing professional athletes in the Euro-League circuit, Moreno-Pérez et al. reported an overall rate of 12.59 injuries per 1000 h played, most of which were found in competition (rather than in training, 77.83 versus 8.29 injuries per 1000 h of play) in the absence of direct contact with another player, in the lower limb, and with greater involvement of the knee. No particular differences were observed between the modalities of onset between training and competition injuries [15].

Several studies published in scientific research proposed the analysis of body composition, such as that carried out through BIVA, as a useful element in the literature on the state of health [16], in the prevention and treatment of pathologies [17], and in the protection of health and in the development of an athlete’s performance [18,19].

All of the studies carried out so far have taken into consideration the use of segmental BIVA in the assessment of asymmetries or in the follow-up of injuries; we have not been able to find evidence of investigations carried out with predictive purposes, particularly in basketball.

The present work aimed to investigate the possibility of predicting injuries and performance status in professional basketball athletes using segmental BIVA analysis.

## 2. Materials and Methods

The experimental work protocol was implemented in the context of the Italian Serie A basketball championship during the 2020–2021 competitive season with the players, medical staff, and technical staff of the US Victoria Libertas Pallacanestro Pesaro (VL Medical Lab powered by Fisioclinics). The working protocol below was set out in accordance with the standards of good clinical practice established by the Declaration of Helsinki and in accordance with the Declaration of the European Union 2001/20/EC [20]. All of the participants provided informed consent to participate in the study, which was approved by the Ethics Committee of the University of Urbino Carlo Bo, Italy (14/2019, date of approval 29 November 2019).

### 2.1. Participants

We looked at fourteen professional male athletes of Caucasian and African–American ethnicity (age 25.5 ± 5.9 years, height 197.7 ± 8.4 cm, weight 94.2 ± 8.4 kg, and BMI 24.1 ± 1.3 kg/m^2^), as presented in Table 1. Since they are professional athletes, medical screening was conducted in all cases, carried out for competitive practice based on protocols established by Italian law in accordance with the Italian Sports Medicine Federation (FMSI) and with the Italian Basketball Federation (FIP) [21,22]. The inclusion criteria included being cleared for medical fitness for competitive physical activity, being actively present in the professional team from the pre-season run-in phase, and participating in at least 80% of the athletic preparation and training phases. The exclusion criteria included the presence of acute pathologies, the presence of pathologies (even resolved) within the last 30 days, previous presence of muscle injuries and/or osteoarticular pathologies in the last 90 days, and recent drug therapies in progress within the last 60 days. All participants provided informed consent to participate in the study, which was approved by the Ethics Committee of the University of Urbino Carlo Bo, Italy (14/2019, date of approval 29 November 2019).

### 2.2. Study Design

The study for the bioimpedance monitoring of the athletes was carried out pre-season (August–October) and in-season (October–May). The pre-season structure (six micro-cycles) involved nine workouts. The weekly micro-cycle generally provided rest and/or conditional–technical work for the day after a match for the athletes who were not very busy during the match, together with physical therapies. In the following days, there were two double sessions per day, alternating micro-cycles of max dynamic force (FMD) with sessions of explosive strength, rapid strength, and specific and technical resistance. In the pre-game, there was a session of joint mobility, tactics, and shooting exercises. During the advanced phase of the in-season period, the weekly load was reduced by providing two single sessions in the infra-weekly period, reducing the training sessions to seven by dedicating more sessions to the game and less to the resistance component. Body composition assessment was performed one time in the pre-season and three times in-season with a single-frequency bioimpedance analysis (SF-BIA), making each assessment both a total body analysis and a segmental analysis. We used a Bioimpedance Analyzer Anniversary 101^®^ model produced by Akern^®^ (Pontassieve, Italy); disposable skin-surface electrodes; Biatrodes^®^, produced by Akern^®^ (Pontassieve, Italy); and Bodygram Dashboard ™ software, produced by Akern^®^ (Pontassieve, Italy), for the evaluation of the derived parameters. Considering the ongoing pandemic during which the 2020/2021 competitive season took place, the assessments in the clinic were also carried out in full compliance with all of the indications provided by the World Health Organization, by the Italian Ministry of Health, by the Italian National Olympic Committee (CONI), by the Italian Basketball Federation (FIP), and by the Italian Sports Medicine Federation (FMSI), as summarized in its guidelines [23]. At each assessment, only the staff necessary in the clinic were present, wearing the individual protection devices (PPE) prescribed by law and proceeding with appropriate sanitization of the environments between one assessment and the following. All of the assessments were performed in a homogeneous time slot compatible with the competitive tasks of the team before training started or before each kind of physical activity, following at least 24 h of rest and complete fasting, excluding the intake of drinks [24]. The detection of anthropometric data (weight, height, and BMI calculations) was carried out according to standard methods, as defined by Ross et al. [25]. The measurement protocol was performed as recently reported by Campa et al. [24], involving positioning the athlete on an adequately sized bed in supine decubitus with the lower limbs abducted by 45° and the upper ones by 30°; cleaning the sites where the disposable skin electrodes were placed with ethyl alcohol; and application of the injector electrodes for the upper limb on the metacarpophalangeal joints, and the sensor electrodes for the upper limb between the distal prominences of the radius and ulna (at a minimum distance of at least 5 cm from the previous electrode). This was followed by the application of the injector electrodes for the lower limb on the metatarsophalangeal joints, and the sensor electrodes for the upper limb between the medial and lateral malleolus of the ankle (at a minimum distance of at least 5 cm from the previous electrode). Then, we verified our assumption of the supine position being at least 10′ (the minimum required, as reported by Campa et al., is 2′). Then, we carried out total body measurements, data recording, and software recording. Next, we performed segmental measurements, data collection, and software recording. Finally, we removed the electrodes, the athlete’s left, and we sanitized the environments and the aids used.

### 2.3. Statistical Analysis

Quantitative variables are expressed as the mean (min–max). To evaluate the dependence of the variables related to injuries, multiple linear regressions with backward stepwise elimination were considered. The dependent variables were the total number of injuries from training (Inj_All), the total duration (days) of suspension due to injuries from training (Dur_Inj_All), the number of injuries from matches (Inf_match), the duration (days) of suspension due to injuries from a match (Dur_Inf_Match), the ratio between the total number of injuries and the duration of all injuries (Dur_Inf_All/Inf_All), plus_minus, the number of assists (Assist), and the number of recoveries (N_rec). The following bio-impedentiometric variables (considering also BMI) were used as predictive factors: global reactance and global resistance (Xc and RZ), trunk reactance and resistance (Xctrunk and RZtrunk); right and left hemisome reactance and resistance (XcEmsDx, RZEmsDx, XCEmsSx, and RZEmsSx); upper and lower hemisome reactance and resistance (XcEmsSup, RZEmsSup, XcEmsInf, and RZEmsInf); right and left arm reactance and resistance (XcArmDx, RZArmDx, XcArmSx, and RZArmSx); and finally, right and left leg reactance and resistance (XcLegDx, RZLegDx, XcLegSx, and RZLegSx). All of the impedentiometric data were obtained and processed with the Bodygram Dashboard ™ software. Stepwise regressions were carried out for multiple regressions several times, each time removing the weakest correlated variable; at the end, the variables that best explained the distribution were reported (*p* of *F* to enter <0.05; *p* of *F* to remove >0.15). The goodness of fit was quantified using R^2^ adjusted, quantifying the % of variability in the dependent variables accounted for by all of the independent variables together. All of the elaborations were performed using Microsoft Excel (Microsoft^®^ Excel^®^ for Microsoft 365 MSO, Version 2206 Build 16.0.15330.20246, 64 bit, Redmond, Washington, USA) and SPSS 22.0 (IBM, Armonk, NY, USA).

## 3. Results

The means and ranges (min–max) of all variables are shown in Table 2.

The results of the multiple linear regression with backward stepwise elimination are presented in Table 3.

Multiple linear regression values were calculated to predict the Inj_all values. A significant regression equation was found (*F*_(1,30)_ = 8.30, *p* = 0.007), with an adjusted R^2^ of 0.191 considering, at the end of the backward stepwise procedure, only one variable. The participants’ predicted CMJ measurements were calculated as follows:

Inj_All = −1.382 + 0.037 × XcEmsSup.

For the Dur_Inj_All variable (*F*_(2,29)_ = 12.94, *p* < 0.001), an adjusted R^2^ of 0.435 was found considering, at the end of the backward stepwise procedure, only two variables. The participants’ predicted Dur_Inj_All measurements were calculated as follows:

Dur_Inj_All = 283.9 − 8.243 × BMI − 2.663 × XcLegDx

For the inf_match variable (F_(2,29)_ = 14.1, *p* < 0.001), an adjusted R^2^ of 0.458 was found considering, at the end of the backward stepwise procedure, only one variable. The participants’ predicted Inf_Match measurements were calculated as follows:

Inf_Match = 0.0038 − 0.087 × XcArmDx.

For the Dur_inf_Match variable (F_(2,29)_ = 12.84, *p* < 0.001), an adjusted R^2^ of 0.433 was found considering, at the end of the backward stepwise procedure, only two variables. The participants’ predicted Dur_inf_Match measurements were calculated as follows:

Dur_inf_Match = 78.93 − 0.799 × RzArmDx − 2.261 × RzTrunk.

For the Plus_Minus variable (F_(1,30)_ = 8.34, *p* = 0.007), an adjusted R^2^ of 0.192 was found considering, at the end of the backward stepwise procedure, only one variable. The participants’ predicted Plus_Minus values were calculated as follows:

Plus_Minus = −148.21 + 0.600 × RzArmDx.

For the Assist variable (F_(2,29)_ = 11.29, *p* < 0.001), an adjusted R^2^ of 0.399 was found considering, at the end of the backward stepwise procedure, only two variables. The participants’ predicted Assist counts were calculated as follows:

Assist = −24.54 + 12.47 × BMI − 1.217 × RzArmDx.

Finally, for the N_rec variable (F_(2,29)_ = 11.07, *p* < 0.001), an adjusted R^2^ of 0.394 was found considering, at the end of the backward stepwise procedure, only two variables. The participants’ predicted N_rec counts were calculated as follows:

N_rec = −295.3 + 9.357 × BMI + 1.521 × XcEsmSx.

## 4. Discussion

The main objective of this study was to investigate the possibility of predicting injuries and the state of performance in professional basketball athletes using segmental BIVA analysis. The study yielded interesting results.

The segmental body composition parameters obtained from BIVA were analyzed to determine a possible positive or negative link with training performance indicators and injury frequency. As these parameters are easily obtainable by quickly trained staff, their variation could be useful for the early prediction of a need for interventions.

The data highlight a positive link between the number of training injuries and XCEmsSup (t = 2.881, *p* = 0.007) and a negative link between training injury duration, and XCLegDx (t = −4.213, *p* < 0.001) and BMI (t = −4.213, *p* < 0.001), suggesting that higher cellularity seems less prone to serious injury during training. Match-day number of injuries negatively correlates with XCArmdx (t = −4.469, *p* < 0.001) and RZArmDx (t = −4.202, *p* < 0.001), while RZTrunk positively correlates with lower match-day injury duration (t = 2.803 *p* = 0.008), in contrast with training data analysis. Analyzing the relationship with performance indicators, RzArmDx has a positive link with plus–minus (t = 2.889, *p* = 0.007) and a negative link with assist number (t = −3.362, *p* = 0.002), contrary to BMI (t *=* 2.254, *p* = 0.032), suggesting a higher level of complexity in match dynamics than in training.

Taking into account training injuries, the data obtained highlight a possible positive link between number of training injuries and upper hemisoma reactance (XCEmsSup); this could be related to a higher upper hemisoma cellularity in subjects with elevated reactance. This finding correlates well with other observed data, which suggest an inverse relationship between training injury duration and higher right lower limb reactance (XCLegDx), and between injury duration and higher body mass index, further highlighting how subjects with higher cellularity seem less prone to severe training injuries.

In partial confirmation of this observation, analyzing neuromuscular movement deficits in 575 young basketball athletes, Adillón et al. reported how the main deficiencies are related to postural and dynamic instability of the lower limb joints, combined with deficits in the jumping/landing technique [26]. More difficult to interpret, and probably in partial contrast, is what Wang and Baek found in the development of a reduction algorithm for early identification of the risk of injuries in basketball. The authors identified, among other factors, the difference between the circumferences of the left and right thighs, while in our study, no correlations emerged for the left lower limb, and the difference in the strength of the anterior and posterior thigh muscles, which cannot be evaluated with the method we used [27]. The lack of evidence for the left lower limb could also be re-evaluated in light of the lack of differences found by Adillón et al. [26] in the stability found between dominant and non-dominant limbs, in accordance with what was previously observed in other studies [28,29].

In contrast, the interpretation of data obtained by analyzing match-day injuries is more challenging. While right upper limb higher reactance (XCArmdx) negatively correlates with match-day number of injuries, as expected, right upper limb resistance (RZArmDx) and trunk resistance (RZTrunk) correlate with lower match day injury duration, in contrast with training data analysis. It is significant that the analysis of the injuries in the match does not show correlations overlapping with what was observed for injuries in training, particularly regarding the parameters referring to the lower limb, as reported by Yeh et al., in which basketball players with a BMI > 25 demonstrated an increased risk of injury [30], a phenomenon also observed in young female players by Shimozaki et al. [31].

Carrying out an analysis of the relationship between the BIVA parameters and performance indicators, we observed how right upper limb resistance (RzArmDx) has a positive link with plus–minus (a sports statistic used to measure a player’s impact on the game), suggesting that higher muscle cellularity of the dominating arm could be an important predictor of player effectiveness. This observation can be considered in light of the findings of Caamaño-Navarrete et al. on the link between muscle mass and capacity for jumping, and speed and ball throw [32].

However, right upper limb resistance negatively correlates with assist number, in opposition to that observed with BMI, which is directly proportional to assist number. Instead, the interpretation of the correlation between left hemisoma reactance (XcEmsSx), BMI and ball possession stats seems not so challenging as both physical parameters positively impact the number of steals. A possible interpretation of these data could be provided in light of what Pojskic et al. observed, identifying among other factors, explosive power as a determinant of the performance capacity of elite basketball players [33]. The development of this knowledge is of considerable importance considering, as observed by Mancha-Triguero et al., the notable lack of specific tests to evaluate athletic qualities in basketball athletes [34].

### 4.1. Future Ideas

The possible future development of these observations undoubtedly requires the acquisition of data from a much larger sample, which allows for an analysis divided on the basis of the roles and physical characteristics of the athletes, with the aim of understanding whether these parameters affect performance. Once this analysis has been carried out, an interesting subsequent development could be that of including the anthropometric parameters in algorithms aimed at predicting the risk of accidents [27] or in a subsequent evolutionary step represented by machine learning systems currently under development [35,36].

### 4.2. Strengths

This study represents the first analysis on professional players in the context of a professional formation of the top flight in the Italian league (in the season of this study, ranked second in the Italian Cup, second tournament for importance of Italian professional basketball teams). This study provides, for the first time, an evaluation of the possibility of developing potentially early predictors of injury and performance. A very important factor is the speed of executing this assessment and the absolute non-invasiveness of the method. Furthermore, these data become particularly interesting considering the robustness of the linear regression on most of the parameters taken into consideration.

### 4.3. Limitations

A significant limitation of this work is represented by the small sample of athletes due to the single team being evaluated. For a better comprehension of the BIVA tool’s potential, it could be very useful to extend this kind of evaluation to a larger number of athletes with comparable characteristics, such as the whole roster of players who compete in Lega Basket Serie A. A further limitation is constituted by the fact that all of the subjects considered are elite athletes, not allowing us to evaluate whether the method is also applicable to other types of populations. This is especially important with regard to the considerations of cell mass, which in other types of subjects, could lead to other types of interpretations.

## 5. Conclusions

This work highlighted how bioelectrical impedance vector analysis, with parameters obtained either from a total body or a segmental analysis, could constitute a valuable tool in athletes’ health maintenance and performance prediction. BIVA could be considered a reliable tool used for retrieving direct and objective parameters that should be used to build a personalized athlete training schedule, thus sorting athletes according to measured data. In addition, BIVA could be a useful tool for preventing injuries or performance loss. Further studies on BIVA and similar applications could provide tools of extreme interest for athlete health and physical integrity preservation, even providing data useful for better understanding athlete performance and for categorizing individual training specificities from the first day on the team using an evidence-based approach.

### Practical Application

These initial results could already be used (with appropriate caution) for further assessments, for example, postural, functional, or structural assessments, carried out with the aim of preventing a possible injury or functionally modifying the training plan of the individual athlete with the intent to support and implement their performance capabilities.

## Figures and Tables

**Table 1:** Age and anthropometric variables of the participants at baseline; mean and standard deviation values for groups are reported.

Parameter	Value	Standard Deviation
Age (year)	25.5	±5.9
Height (cm)	197.7	±8.4
Weight (kg)	94.2	±8.4
BMI (kg/m^2^)	24.1	±1.3

**Table 2:** Average, minimum, and maximum values for dependent and independent variables analyzed in the 14 professional basketball players.

Variables		Mean (Min–Max)
*Dependent variables*	Inj_All	0.66 (0–3)
	Dur_Inj_All (days)	7.19 (0–60)
	Dur_Inj_All/Inj_All (days)	6.23 (0–60)
	Inf_Match	0.21 (0–1)
	Dur_inf_Match (days)	9.16 (0–60)
	Plus_Minus	−36 (−81–3)
	Assist	44.5 (1–185)
	N_rec	18.75 (1–62)
*Independent variables*	BMI (kg/m^2^)	23.8 (21.3–26.0)
	Xc (Ω)	61.4 (47–72)
	Rz (Ω)	429 (367–632)
	XcTrunk	6.3 (4–8)
	RzTrunk	35.5 (18–44)
	XcEmsDx	59.5 (47–69)
	RzEmsDx	422 (377–485)
	XcEmsSx	60.1 (50–69)
	RzEmsSx	422.7 (377–485)
	XcEmsSup	54.7 (43–102)
	RzEmsSup	376 (300–436)
	XcEmsInf	60.1 (45–73)
	RzEmsInf	432 (377–484)
	XcArmDx	26.4 (18–31)
	RzArmDx	187 (143–215)
	XcArmSx	26.8 (19–31)
	RzArmSx	188.4 (154–229)
	XcLegDx	30.3 (22–38)
	RzLegDx	215.9 (188–242)
	XcLegSx	30.3 (18–36)
	RzLegSx	217.0 (185–245)

**Table 3:** Multiple linear regression analysis with gradual backward elimination of the examined variables in the 14 professional basketball players.

Dependent Variable	Independent Variable	Standardized β	Standard Error	t Value	*p* > |t|
Inj_All	XcEmsSup	0.037	0.013	2.881	0.007
Dur_Inj_All	BMI	−8.243	1.817	−4.536	<0.001
	XcLegDx	−2.663	0.632	−4.213	<0.001
Inf_Match	XcArmDx	−0.087	0.019	−4.469	<0.001
Dur_inf_Match	RzArmDx	−0.799	0.191	−4.202	<0.001
	RzTrunk	2.261	0.787	2.873	0.008
Plus_Minus	RzArmDx	0.601	0.208	2.889	0.007
Assist	BMI	12.47	5.532	2.254	0.032
	RzArmDx	−1.217	0.362	−3.362	0.002
N_rec	BMI	9.358	2.051	4.564	<0.001
	XcEmsSx	1.521	0.502	3.042	0.005

## Data Availability

Not applicable.
